# Naltrexone during pain conditioning: A double-blind placebo-controlled experimental trial

**DOI:** 10.1177/1744806920927625

**Published:** 2020-06-14

**Authors:** Moa Pontén, Jens Fust, Eva Kosek, Joar Guterstam, Karin Jensen

**Affiliations:** 1 Department of Clinical Neuroscience, Karolinska Institutet, Stockholm, Sweden; 2 Centre for Psychiatric Research, Karolinska Institutet, Stockholm, Sweden

**Keywords:** Pain conditioning, pressure pain, opioid antagonist, endogenous opioids

## Abstract

Naltrexone reversibly blocks the effects of opioids and has been shown to decrease placebo analgesia. However, it is not clear (1) to what extent naltrexone affects pain modulation in a nontreatment context, for example, in response to pain cues or (2) how naltrexone given prior to pain-cue learning shapes pain responses. In a double-blind procedure prior to pain-cue conditioning, 30 healthy participants were randomized to receive an oral dose of naltrexone (50 mg) or inert pill. During functional magnetic resonance imaging, high and low pain pressures were paired with two different visual cues: a high pain cue and a low pain cue (learning sequence). During a test sequence, medium levels of pressure were used for both cues and the difference in subjective pain ratings following high and low pain cues was calculated. Results showed significant conditioned pain responses across groups (*P *<* *.001); however, no significant difference between participants receiving naltrexone or inert pill (*P *=* *.193). There was a significant correlation between the difference in high and low pain ratings during the learning sequence and the effect of high and low pain cues during the test sequence (r = .575, *P *=* *.002). Functional magnetic resonance imaging analyses revealed no significant difference in brain activation between groups. Here, we demonstrate comparable learning of pain responses in participants treated with naltrexone or inert pill. The results point to the possibility that associative learning, and conditional responding to pain cues, is not dependent on endogenous opioids. Our results, using pain-cue conditioning to create reduced pain responses, contrast previous studies where opioid antagonists significantly reduced the placebo effect in treatment of pain.

## Introduction

Endogenous opioids have been demonstrated to play an important role in pain modulation.^1,2^ For the last four decades, the opioid antagonist in the form of an injection (naloxone) or pill (naltrexone) has been used to investigate the role of endogenous opioids in different experiments using different pain modalities.^3–7^

A seminal study by Levine et al.^6^ demonstrated a partial block of placebo responses in postoperative patients following administration of naloxone. Patients who were responders to placebo analgesia reported increased pain levels after administration of naloxone which suggest that endogenous opioids mediate placebo analgesia. Since then, the role of endogenous opioids in placebo analgesia has been demonstrated in several studies;^3,4,8,9^ for a review, see Sauro and Greenberg.^10^

However, the evidence is inconclusive as there are studies that have found no blocking effect of naloxone on placebo analgesia,^11–13^ whereas other studies have shown partial blockage of the placebo analgesic response.^3,4^ The neurobiology underlying placebo analgesic responses seems to be a flexible system involving different mechanisms and neurotransmitters,^14^ and it is imperative to discern in what contexts placebo analgesia is blocked by naloxone and what mechanisms are naloxone-insensitive.^15^

In a typical experiment investigating endogenous opioids in placebo analgesia, the experiment includes some sort of placebo treatment to establish a placebo response. Then, placebo responses already established, participants are given the opioid antagonist and the placebo analgesic effect is measured again.

Classical conditioning is often used to investigate placebo mechanisms, where an initially neutral stimulus (conditioned stimulus) is associated with an unconditioned stimulus that leads to an appetitive or aversive outcome. This can be combined with verbal information.^16^ Classical conditioning does not represent placebo analgesia per se, as it does not contain any treatment. However, classical conditioning is a core mechanism of placebo analgesia^17^ and can affect sensory perception, as demonstrated by decreased pain in several studies.^18,19^

The aim of this study was to examine the role of endogenous opioids in response to conditioning of cues that signal either high pain or low pain. The conditioned cues consisted of the words “high” or “low” and were thus not neutral. A randomized, double blind, placebo-controlled experiment with parallel groups was performed where the opioid antagonist naltrexone or placebo (inert pill) was given prior to pain conditioning in a functional magnetic resonance imaging (fMRI) scanner. The hypothesis was that participants randomized to naltrexone would display lower conditioned pain responses (i.e., lower cue effect) than participants in the inert pill group. For clarity we report fMRI data, however, this study was underpowered and therefore no hypothesis was formulated a priori.

## Method

A total of 30 healthy male volunteers were randomized in a double-blind fashion to receive an oral dose of either naltrexone or inert pill. Groups did not differ regarding age or pain sensitivity. Inclusion criteria were as follows: male, right-handed, age between 20 and 55 years. Exclusion criteria were as follows: history of medical or psychiatric illness, ongoing medication for any chronic illness or psychiatric illness, metal implants, claustrophobia, or any other counter indication for MRI. Two participants were excluded due to technical failure and one due to claustrophobia resulting in data from 27 healthy male volunteers (mean age: 40.3 years, standard deviation (SD) = 8.3; education level: higher education 73%, high school 27%) included in the final analysis. Participants were recruited as healthy controls in a larger study investigating the effects of naltrexone on cue reactivity and craving in amphetamine dependence.^20^ Recruitment was performed via advertisements. The regional Ethics Review Board in Stockholm approved the advertisements and the study (Dnr: 2012/1062/32), and all participants gave written informed consent. The participants were debriefed after study end and informed they could withdraw their data.

### Material

Painful stimulations were applied using an automated, pneumatic, computer-controlled stimulator with a plastic piston that applied pressure pain to the left thumb nail via a 1 cm^2^ hard rubber probe.^21^ The software presentation (Neurobehavioral Systems, Neurobs.com) was used for presentations of the images in the magnetic resonance (MR) scanner.

### Procedure

Participants were screened for inclusion and exclusion criteria by a physician and then scheduled for an experiment. Participants were informed that the experiment investigated “the brain activity during high and low pressures,” but the full purpose of the study was not revealed until the experiment was over.

#### Pharmacological challenge

Each participant left a urine sample before taking the medication to exclude current use of opioids. All participants showed negative results. Approximately 1 h before the experiment, in a randomized double-blind procedure, participants received an oral dose of either naltrexone 50 mg (n = 14) or inert pill (n = 13). The dose of 50 mg naltrexone has previously shown reliable effects in studies of pain modulation.^7^ A single dose of 50 mg of naltrexone is enough for a near complete blockade of the endogenous µ opioid system.^22–24^

#### Calibration

The participants were calibrated for subjective pain ratings by receiving ascending series of pressure stimuli. Participants were instructed to rate the intensity of the pain on a 0 to 20 scale (Gracely scale) by pressing on a rating device. The pressures were presented in steps of 50 kPa to determine the pressure pain threshold (first pain rating >0) and stimulation high pressure pain (first pain rating >15). These levels were then used to calculate and test three pressures in between the threshold and the high pressure pain. The duration of each pressure was 2.5 s. Based on the individual ratings of the different calculated pressures, each participant’s approximate low pressure pain (5 Gracely) and high pressure pain (15 Gracely) were determined and tested for consistency in ratings.

#### Experimental paradigm

After this, participants were tested with the visual paradigm. Pictures were presented inside the MR scanner via visual goggles (binocular organic light emitting diode display; Nordic Neuro Lab, Norway). Participants were informed that the pain cue (text on the screen “HIGH” or “LOW” pain) indicated the subsequent pressure.

During the learning sequence, pressure pain stimuli were applied to left thumb with randomized time intervals so that the timing between stimuli could not be predicted. The participants were asked to focus on a cross in the middle of the screen. Before the onset of each painful stimulus, the word “HIGH” or “LOW” would appear on the screen instead of the cross, indicating that a high or low pressure would soon follow. The duration of the visual cue (“HIGH” or “LOW”) was 2 s, followed by a jittered wait (2–6 s) before the painful pressure onset. After the painful pressure, a pain rating scale (0–20 Gracely scale) appeared after a jittered wait (2–6 s). The learning sequence consisted of 20 trials and all pressures lasted 2.5 s.

The learning sequence was directly followed by a test sequence in which the pain cues (“HIGH” or “LOW”) were always followed by a medium pressure in between each participants’ calibrated high and low pressure. The test sequence consisted of 20 trials, where 10 stimuli were preceded by a “HIGH” cue and 10 stimuli by a “LOW” cue.

#### fMRI protocol

The experiment was performed in a 3T General Electric 750 MR scanner using an eight-channel head coil at MR Research Center, Karolinska Institutet, Stockholm. Whole brain volumes were acquired using a T2*-weighted single-shot gradient echo planar imaging sequence. The following parameters were used: repetition time (TR)/echo time (TE) = 2000/30 ms, flip angle = 70°, field of view = 220 × 220 mm, matrix size = 72 × 72, 42 slices, slice thickness = 3mm with a 0.5 mm gap, acquired through an interleaved slice acquisition mode. Anatomical MR scans were acquired with a high-resolution brain volume imaging (BRAVO) three-dimensional T1-weighted image sequence (1 × 1 × 1 mm voxel size, 176 slices). Anatomical (T2-weighted) scans were investigated by a neuroradiologist for clinical abnormalities. Earplugs and cushions were used to dampen scanner noise and reduce head movement.

### Data analysis

Statistical analyses of behavioral data were conducted using R® and SPSS version 24 (IBM) for correlations, demographics, and figures.^25^ The threshold for statistical significance was set at *P *<* *.05, and all tests were two-tailed. fMRI data analyses were performed using the Statistical Parametric Mapping 12 (SPM12) software using Matlab2014 (The MathWorks, Inc., MA, USA). Functional images were preprocessed with default settings in SPM12. Images were spatially realigned, coregistered, and normalized to the Montreal Neurological Institute space and spatially smoothed using an 8 mm full-width-half-maximum Gaussian kernel. First-level general linear model was built on the regressors: high pain-cue anticipation and high pain-cue pain as well as low pain-cue anticipation and low pain-cue pain. High pain-cue and low pain-cue anticipations refer to the jittered wait before pressure onset during the learning sequence.

An a priori power analysis was performed to determine the sample size required to detect a pain-cue effect (n = 13) based on a previous data set with similar design.^18^ Calculations were performed in G*Power (3.1) based on differences in pain ratings (0–100) between two pain cues (dependent means) M = 17, SD of difference = 13, alpha = .05, power (1 − β) =.99, two-tailed.

#### Baseline characteristics

Demographic characteristics for all participants were retrieved concerning age and education. Mean and SDs for pain thresholds, high pressure pain, and test-sequence medium pressure were calculated (see Table 1).

**Table 1.** Baseline characteristics.

Group	Number of participants	Pain threshold (kPa)Mean (SD)	High pain (kPa) Mean (SD)	Medium pain during test sequence (kPa)Mean (SD)
Inert pill	*n* = 13	250 (114.81)	515.38 (128.1)	380.77 (108.1)
Naltrexone	*n* = 14	182.14 (72.34)	517.86 (143.59)	344.71 (102.05)

Note: Pain threshold refers to the first rated pressure stimulation above 0 (0–20 Gracely scale), high pain refers to the first rated pressure stimulation above 15 (0–20 Gracely scale). Medium pain refers to the pressure for the test sequence. Pressure pain was given to the thumb nail using a 1 cm^2^ piston in hand-held pneumatic pressure pain device. Pressure units are given in kPa and represent the group average of calibrated threshold, high pain, and the calculated medium pain used during the test sequence. SD: standard deviation.

#### Pain ratings

Pain ratings collected during the test sequence were modeled using a linear mixed effect analysis due to the hierarchical structure of the data. The model was used to assess the influence of the interaction between condition (naltrexone/inert pill) and cue (“HIGH”/”LOW”) on pain ratings. As random effects, we used by-subject random intercept and by-subject random slopes for trial and cue. Post hoc correlation analyses (Pearson correlation) were done to investigate the relationship between pain ratings during the learning sequence and test sequence as well as the relationship between pain sensitivity and pain ratings during test sequence.

The data sets generated during and analyzed during this study are available from the corresponding author on reasonable request.

## Results

**Figure 1.**
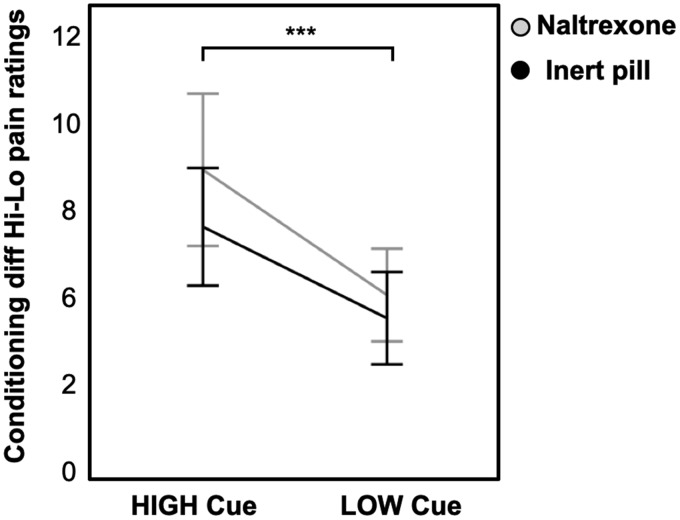
Visual representation of mean pain ratings (rated on the 0–20 Gracely scale) during the test sequence. There were significant conditioned pain responses in the naltrexone group (*P < *.001) and inert pill group (*P < *.001). Error bars represent two standard errors.

Participants in the naltrexone and inert pill groups were comparable regarding baseline measures of pain sensitivity (medium pain value used during pain testing, see Table 1). There were significant conditioned pain responses across groups, linear: *b* = −5.99*, t*(39) = −8.18, *P < *.001 (see Figure 1) indicating that the high cue was followed by relatively higher pain ratings compared to the low cue, in spite of identical absolute pressures during the test sequence. There was no significant difference in pain perception during the test sequence between participants receiving naltrexone or inert pill, linear: *b =* 1.39, *t*(39) = 1.32, *P *=* *.193. Looking at the groups separately, in a post hoc analysis, there were significant conditioned pain responses in both the naltrexone group (*b* = −3.61*, t*(14) =−4.5, *P < *.001) and the inert pill group (*b* = −2.67*, t*(13) = −5.55, *P < *.001).

**Figure 2.**
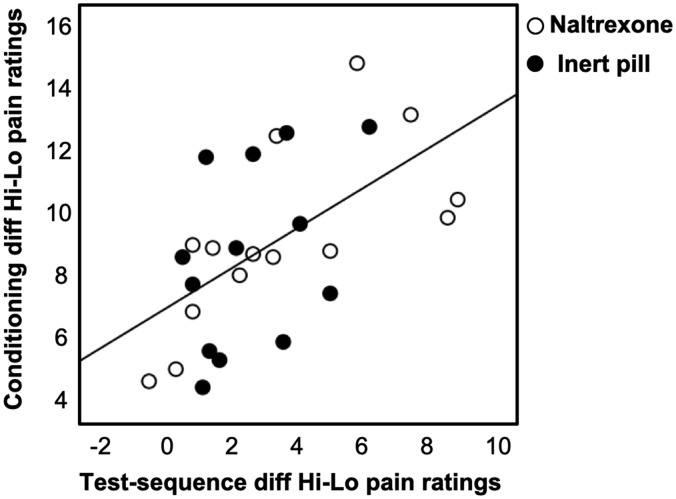
Scatter plot illustrating the positive correlation between the difference in ratings (high pain–low pain) during conditioning (strength of learning) and ratings (high cue − low cue) during the test sequence; r = 0.575, *P *=* *.002.

Pain sensitivity did not explain the variance in pain ratings during the test sequence, as the pressure required to induce high pain (kPa) did not correlate with pain ratings in response to the high cue (r = .021, *P *=* *.918). Conversely, the pressure required to induce low pain did not correlate with low cue pain ratings (r = −.079, *P *=* *.699) during the test sequence. This indicates that baseline differences in pain sensitivity did not explain the variance in test-sequence pain ratings. However, there was a positive correlation between the difference in ratings (high pain–low pain) during the learning sequence and ratings (high cue–low cue) during the test sequence; r = .575, *P *=* *.002 (see Figure 2), indicating that the learning that takes place during conditioning (i.e., perceived difference between high and low pain) may explain the pain perception during the test sequence.

**Figure 3.**
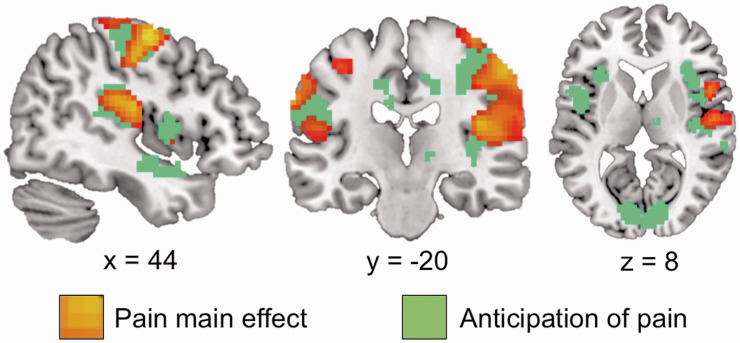
Representation of increased blood oxygenation level dependent (BOLD) signal in pain relevant cortical areas during pressure pain and anticipation for pain.

Analyses of brain activations in response to pain during the learning sequence (across groups) revealed activations in pain processing regions (high pain > low pain) such as primary somatosensory cortex (S1), supplementary motor area and secondary somatosensory cortex (S2), as well as posterior insula (see Figure 3). Yet, there were no significant effects of the main experimental comparisons, for example, low cue–high cue across groups and no differences between groups either (see Table 2).

**Table 2.** Results from fMRI statistical analyses of *pain* activations and *pain anticipation* across both groups during the learning phase.

	MNI x	MNI y	MNI z	Cluster size (voxels)	Z score	FWE P value
Pain main effect (high pain > low pain)						
S1	57	−10	50	239	5.43	.001
SMA/ACC	6	−1	56	245	4.85	.009
S2/posterior insula	54	−13	14	181	4.63	.023
Anticipation of pain main effect (high pain cue > low pain cue)						
S1/SMA/ACC	42	−4	62	693	4.77	.020

Note: Pain main effect refers to the brain activations in response to pressure pain during the learning phase (high pain > low pain). Coordinates (x, y, z) correspond to the MNI standard brain atlas. Statistical threshold was set at *P *<* *.001, reported clusters are FWE-corrected at the cluster level. Anticipation to pain main effect refers to the brain activations in response to the anticipation of pressure pain during the learning phase (high pain cue > low pain cue). S1: primary somatosensory cortex; SMA: supplementary motor area; ACC: anterior cingulate cortex; S2: secondary somatosensory cortex; FWE: family-wise error; MNI: Montreal Neurological Institute.

## Discussion

Here, we investigated the role of endogenous opioids in pain conditioning using naltrexone and conditioning of cues that signal either high or low pain. There was a significant conditioned pain effect; however, no significant differences between the group receiving naltrexone or inert pill. Our findings point to the possibility that conditional responding to pain cues is not dependent on endogenous opioids, as there were significant conditioned pain effects both in individuals on naltrexone and inert pill. One unique aspect of our study was the administration of naltrexone (or inert pill) already before the conditioning procedure. Other studies^3,6^ have administered the opioid antagonist in a later stage, when the acquisition of conditioned effects have already been formed. While we found significant effects of the conditioning procedure, our data indicate that pain conditioning is possible with an opioid antagonist on board and does not rely solely on opioid mechanisms.

Based on evidence from experiments on placebo analgesia, demonstrating that opioid antagonists block (at least partly) the analgesic effects,^3,4,8^ it is likely that conditioned pain relief also depends on the release of endogenous opioids. Brain imaging studies have provided further evidence for the involvement of mu opioids during placebo analgesia^26^ and brain areas rich in mu opioid receptors are activated during placebo analgesia.^2,27^ At the same time, studies indicate that numerous factors are involved in placebo effects and endogenous opioids may not always be the key mechanism.^11,28^

Previous data suggest that pharmacological conditioning of analgesic responses can be naloxone insensitive if the conditioning is performed with a nonopioid analgesic.^8^ If verbal suggestion is involved, a part of the analgesic effect can be reversible with naloxone. Even if this study did not involve pharmacological conditioning, the study by Amanzio and Benedetti^8^ points to the possibility that some conditioned pain effects are not dependent on opioids (unless there is direct conditioning with an opioid drug). One more study confirms that type of pharmacological conditioning determines whether naloxone will affect pain ratings.^28^ Nonopioid pharmacological conditioning with nonsteroidal anti-inflammatory drugs was not reversed by naloxone but with an endocannabinoid antagonist. In line with our study, this suggests that not all placebo-like effects on pain are reversible by naloxone, and that the drug used during conditioning will have effect on the neurobiological mechanisms during placebo analgesia, as if placebo is mimicking the effect of the active drug.

Another reason why the effect of opioid antagonists on placebo analgesia may vary is the difference between study populations. The authors of a clinical trial in patients with irritable bowel syndrome (IBS) suggest that the clinical benefits from placebo treatment are likely not mediated by opioids as naloxone did not affect the outcomes in IBS patients.^11^ Taken together, it is likely that a number of different neurotransmitters can be involved in placebo analgesia^14,28^ and possibly also in conditioned pain responses, and that the response may vary depending on context.

We performed the naltrexone administration *before* the learning of pain relief as opposed to after. This distinction is important as endogenous opioids have been proposed to be involved in learning of responses, such as threat.^29^ For example, administering naloxone before learning of conditioned fear responses has shown to enhance acquisition of conditioned fear.^30^ To the best of our knowledge, studies on placebo analgesia to date have all administered naltrexone *after* the learning of placebo analgesic responses.

We employed a stimulus context where pain modulation was dependent on predictions of stimulus intensities, and not the efficacy of a given treatment. The studies that have successfully blocked or partially blocked the placebo response have all been performed in a so-called treatment context. Although the mechanisms are thought to overlap, preliminary evidence suggest different brain circuitries involved in pain relief from stimulus expectancy and treatment expectancy.^19^ Here we used conditioning with two different visual cues, one associated with high pain (“HIGH”) and one associated with low pain (LOW”). As our conditioning paradigm did not involve any treatment, and represents a typical response expectancy paradigm, we suggest that it may involve a nonopioid mechanism that will not be affected by opioid blockade.

The lack of naloxone effect on pain modulation in previous studies has also been discussed in terms of dosage, where authors speculate that the dose might have been too low to have an effect.^11,31^ Here we used a single dose of 50 mg naltrexone. Previous studies of fear conditioning in humans used the same dose,^29^ and a pain study showed that 50 mg naltrexone is enough to successfully block pain inhibition^7^ as well as studies showing almost complete blockade of endogenous opioid system with the same dose.^22–24^ Hence the dose in this study was likely not too low to exert an effect.

Naltrexone and naloxone are two unselective opioid antagonists with similar pharmacodynamic profile and differ mainly in terms of pharmacokinetics, where naloxone has a shorter half-life and poor oral bio-availability. This study used oral naltrexone, as it was part of a larger project investigating this as a potential treatment for amphetamine addiction. For the purposes of this experiment, the resulting pharmacological effect of a strong opioid blockade would be similar with naloxone.

We found a correlation between the difference in pain ratings in response to high and low pain during the learning sequence and the difference between pain ratings in response to high and low cues during the test sequence. This indicates that early learning of an association predicts subsequent conditioned effects. This is in line with the Jensen et al. study, using a similar conditioning paradigm, where the strength of learning predicted subsequent placebo and nocebo effects.^18^ The role of learning in the acquisition of placebo and nocebo effects is further emphasized in a study by Benedetti et al.^33^ where the number of conditioning trials had a linear relationship to the firing of thalamic neurons during placebo treatment, and clinical benefits. This study suggests that associative learning has a significant effect on pain in healthy volunteers, even if naltrexone has blocked the function of the opioid system.

This study was limited by the small sample size and further studies are needed to confirm these preliminary findings. One possibility could have been to use Bayesian statistics in order to fully claim that lack of effect means no group differences. However, those analyses were not planned and we were not powered enough to do such analyses. Despite this, our findings add to previous placebo studies showing none to limited effect of opioid antagonist on pain relief.^11,12,32^

This study could not determine if there is a difference between placebo-like responses in a stimulus context (e.g., pain-cue effects) or treatment context (e.g., placebo pill) as we only tested the effects of naltrexone in a stimulus context. In addition, as the conditioning included cues that signaled either high or low pain it was not possible to test if the responses was mainly attributable to automatic learning processes or explicitly formed predictions.

In contrast to previous studies, we used pressure pain, as it has been shown to evoke a more clinically relevant pain sensation (that also correlates to clinical pain measures).^34^ It is possible that a different pain modality would have rendered a different result in this study. However, our model of experimental pain produced robust placebo responses and was not affected by naltrexone. Pressure pain has in addition been shown to be unaffected by naloxone.^35^

This study included fMRI analyses, yet, there were few significant findings. Overall, we found significant neural activations in pain relevant areas during pressure stimuli, and anticipation of pressure stimuli. Yet, our analyses failed to find significant activations for the high cue and low cue condition during the test sequence, or differences between the naltrexone and placebo group. The lack of fMRI findings is likely due to low power as our groups only had 14 and 13 participants, respectively. The fMRI data was included for exploratory reasons and included in this article to provide the reader with the full picture of the data collected. Due to the exploratory nature of the fMRI data we did not form specific hypotheses a priori.

In conclusion, comparable conditioned pain responses were shown for participants in the naltrexone and inert pill group. This points to the possibility that the full function of the endogenous opioids is not necessary for conditioned pain responding. Our results imply that the neurobiological system of pain relief is flexible and consists of different mechanisms depending on the experimental context. The findings in this study may further deepen the knowledge about the role of endogenous opioids in conditioning and may be of clinical importance where knowledge of basic pain processing is key. Further research is needed to investigate the neurobiological mechanisms of conditioned pain responses and placebo analgesia.

## References

[1] Scott DJ Stohler CS Egnatuk CM Wang H Koeppe RA Zubieta JK. Placebo and nocebo effects are defined by opposite opioid and dopaminergic responses. Arch Gen Psychiatry 2008; 65: 220–231. 18250260 10.1001/archgenpsychiatry.2007.34

[2] Wager TD Scott DJ Zubieta JK. Placebo effects on human mu-opioid activity during pain. Proc Natl Acad Sci USA 2007; 104: 11056–11061. 17578917 10.1073/pnas.0702413104 PMC1894566

[3] Eippert F Bingel U Schoell ED Yacubian J Klinger R Lorenz J Büchel C. Activation of the opioidergic descending pain control system underlies placebo analgesia. Neuron 2009; 63: 533–543. 19709634 10.1016/j.neuron.2009.07.014

[4] Grevert HP Albert HL Goldstein HA. Partial antagonism of placebo analgesia by naloxone. Pain 1983; 16: 129–143. 6308540 10.1016/0304-3959(83)90203-8

[5] Levine JD Gordon NC. Influence of the method of drug administration on analgesic response. Nature 1984; 312: 755–756. 6514008 10.1038/312755a0

[6] Levine JD Gordon NC Fields HL. The mechanism of placebo analgesia. Lancet 1978; 2: 654–657. 80579 10.1016/s0140-6736(78)92762-9

[7] King CD Goodin B Kindler LL Caudle RM Edwards RR Gravenstein N Riley JL 3rd Fillingim RB. Reduction of conditioned pain modulation in humans by naltrexone: an exploratory study of the effects of pain catastrophizing. J Behav Med 2013; 36: 315–327. 22534819 10.1007/s10865-012-9424-2 PMC3774309

[8] Amanzio M Benedetti F. Neuropharmacological dissection of placebo analgesia: expectation-activated opioid systems versus conditioning-activated specific subsystems. J Neurosci 1999; 19: 484–494. 9870976 10.1523/JNEUROSCI.19-01-00484.1999 PMC6782391

[9] Benedetti F. The opposite effects of the opiate antagonist naloxone and the cholecystokinin antagonist proglumide on placebo analgesia. Pain 1996; 64: 535–543. 8783319 10.1016/0304-3959(95)00179-4

[10] Sauro MD Greenberg RP. Endogenous opiates and the placebo effect: a meta-analytic review. J Psychosom Res 2005; 58: 115–120. 15820838 10.1016/j.jpsychores.2004.07.001

[11] Vase EL Robinson GM Verne DN Price DD. Increased placebo analgesia over time in irritable bowel syndrome (IBS) patients is associated with desire and expectation but not endogenous opioid mechanisms. Pain 2005; 115: 338–347. 15911161 10.1016/j.pain.2005.03.014

[12] Posner J Burke CA. The effects of naloxone on opiate and placebo analgesia in healthy-volunteers. Psychopharmacology (Berl) 1985; 87: 468–472. 2867576 10.1007/BF00432515

[13] Gracely RH Dubner R Wolskee PJ Deeter WR. Placebo and naloxone can alter post-surgical pain by separate mechanisms. Nature 1983; 306: 264–265. 6646208 10.1038/306264a0

[14] Buchel C Geuter S Sprenger C Eippert F. Placebo analgesia: a predictive coding perspective. Neuron 2014; 81: 1223–1239. 24656247 10.1016/j.neuron.2014.02.042

[15] Fields HL Levine JD. Placebo analgesia – a role for endorphins? Trends Neurosci 1984; 7: 271–273.

[16] Carlino E Torta DM Piedimonte A Frisaldi E Vighetti S Benedetti F. Role of explicit verbal information in conditioned analgesia. Eur J Pain 2015; 19: 546–553. 25161110 10.1002/ejp.579

[17] Wager TD Atlas LY. The neuroscience of placebo effects: connecting context, learning and health. Nat Rev Neurosci 2015; 16: 403–418. 26087681 10.1038/nrn3976 PMC6013051

[18] Jensen KB Kaptchuk TJ Kirsch I Raicek J Lindstrom KM Berna C Gollub RL Ingvar M Kong J. Nonconscious activation of placebo and nocebo pain responses. Proc Natl Acad Sci USA 2012; 109: 15959–15964. 23019380 10.1073/pnas.1202056109 PMC3465419

[19] Schenk LA Sprenger C Onat S Colloca L Buchel C. Suppression of striatal prediction errors by the prefrontal cortex in placebo hypoalgesia. J Neurosci 2017; 37: 9715–9723. 28883019 10.1523/JNEUROSCI.1101-17.2017 PMC5628411

[20] Guterstam J Jayaram-Lindstrom N Berrebi J Petrovic P Ingvar M Fransson P Franck J. Cue reactivity and opioid blockade in amphetamine dependence: a randomized, controlled fMRI study. Drug Alcohol Depend 2018; 191: 91–97. 30096639 10.1016/j.drugalcdep.2018.06.023

[21] Jensen BK Kosek CRE Petzke CRF Carville CRS Fransson CRP Marcus CRH Williams CRS Choy CRE Giesecke CRT Mainguy CRY Gracely CRR Ingvar C. Evidence of dysfunctional pain inhibition in fibromyalgia reflected in rACC during provoked pain. Pain 2009; 144: 95–100. 19410366 10.1016/j.pain.2009.03.018

[22] Preston KL Bigelow GE. Differential naltrexone antagonism of hydromorphone and pentazocine effects in human volunteers. J Pharmacol Exp Ther 1993; 264: 813–823. 7679737

[23] Lee MC Wagner HN Jr Tanada S Frost JJ Bice AN Dannals RF. Duration of occupancy of opiate receptors by naltrexone. J Nucl Med 1988; 29: 1207–1211. 1988/07/01. 2839637

[24] Schuh KJ Walsh SL Stitzer ML. Onset, magnitude and duration of opioid blockade produced by buprenorphine and naltrexone in humans. Psychopharmacology (Berl) 1999; 145: 162–174. 10463317 10.1007/s002130051045

[25] R Core Team R. A language and environment for statistical computing. Vienna: R Foundation for Statistical Computing, 2019.

[26] Zubieta J-K Bueller JA Jackson LR Scott DJ Xu Y Koeppe RA Nichols TE Stohler CS. Placebo effects mediated by endogenous opioid activity on {micro}-opioid receptors. J Neurosci 2005; 25: 7754–7762. 16120776 10.1523/JNEUROSCI.0439-05.2005 PMC6725254

[27] Petrovic P Kalso E Petersson KM Ingvar M. Placebo and opioid analgesia – imaging a shared neuronal network. Science 2002; 295: 1737–1740. 11834781 10.1126/science.1067176

[28] Benedetti F Amanzio M Rosato R Blanchard C. Nonopioid placebo analgesia is mediated by CB1 cannabinoid receptors. Nat Med 2011; 17: 1228–1230. 21963514 10.1038/nm.2435

[29] Haaker J Yi J Petrovic P Olsson A. Endogenous opioids regulate social threat learning in humans. Nat Commun 2017; 8: 15495. 28541285 10.1038/ncomms15495 PMC5458514

[30] Eippert F Bingel U Schoell E Yacubian J Büchel C. Blockade of endogenous opioid neurotransmission enhances acquisition of conditioned fear in humans. J Neurosci 2008; 28: 5465–5472. 18495880 10.1523/JNEUROSCI.5336-07.2008 PMC6670627

[31] Price DD Mayer DJ. Evidence for endogenous opiate analgesic mechanisms triggered by somatosensory stimulation (including acupuncture) in humans. Pain Forum 1995; 4: 40–43.

[32] Skjelbred P, Lokken P. Effects of naloxone on post-operative pain and steroid-induced analgesia. *Br J Clin Pharmacol* 1983; 15: 221–226. 10.1111/j.1365-2125.1983.tb01489.x PMC1427855 6849755

[33] Benedetti F Frisaldi E Carlino E Giudetti L Pampallona A Zibetti M Lanotte M Lopiano L. Teaching neurons to respond to placebos. J Physiol (Lond) 2016; 594: 5647–5660. 26861164 10.1113/JP271322 PMC5043026

[34] Geisser ME Gracely RH Giesecke T Petzke FW Williams DA Clauw DJ. The association between experimental and clinical pain measures among persons with fibromyalgia and chronic fatigue syndrome. Eur J Pain 2007; 11: 202–207. 16546424 10.1016/j.ejpain.2006.02.001

[35] Hermans L Nijs J Calders P De Clerck L Moorkens G Hans G Grosemans S Roman De Mettelinge T Tuynman J Meeus M. Influence of morphine and naloxone on pain modulation in rheumatoid arthritis, chronic fatigue syndrome/fibromyalgia, and controls: a double-blind, randomized, placebo-controlled, cross-over study. Pain Pract 2018; 18: 418–430. 28722815 10.1111/papr.12613

